# Prognostic nomogram that predicts progression-free survival and overall survival of patients with ovarian clear cell carcinoma

**DOI:** 10.3389/fonc.2022.956380

**Published:** 2022-10-31

**Authors:** Jiayi Li, Dongyan Cao

**Affiliations:** ^1^ Department of Nuclear Medicine, Peking Union Medical College Hospital, Chinese Academy of Medical Sciences & Peking Union Medical College, Beijing, China; ^2^ Department of Obstetrics and Gynecology, Peking Union Medical College Hospital, Chinese Academy of Medical Sciences & Peking Union Medical College, Beijing, China; ^3^ National Clinical Research Center for Obstetric & Gynecologic Diseases Department of Obstetrics and Gynecology, Peking Union Medical College Hospital, Chinese Academy of Medical Sciences & Peking Union Medical College, Beijing, China

**Keywords:** nomogram, ovarian clear cell carcinoma, progression-free survival, overall survival, predictive model

## Abstract

**Objectives:**

We aims to develop nomograms to predict progression-free survival (PFS) and overall survival (OS) in patients with ovarian clear cell carcinoma (OCCC) after primary treatment and compare the predictive accuracy with the currently used International Federation of Gynecology and Obstetrics (FIGO) system.

**Methods:**

We collected data from 358 Chinese patients diagnosed with OCCC and who underwent standard treatment at our hospital. Patients diagnosed from 1982-9 to 2011-12 were classified as the training group and patients diagnosed from 2012-1 to 2016-11 were classified as the validation group. Nomograms were developed based on the training group and was validated in the validation group. The predictive performance was determined by concordance index and calibration curve.

**Results:**

The most predictive nomogram for PFS was constructed using variables: thrombosis, the FIGO staging, residual of the tumor and distant metastasis, with a concordance index of 0.738. While the nomogram for OS consisted of thrombosis, lymph node metastasis, residual of the tumor, malignant ascites/washing, and platinum resistance, with a concordance index of 0.835. The nomograms were internally validated by concordance indexes of 0.775 and 0.807 for predicting PFS and OS, respectively. In comparison, the concordance statistics for OS based on the FIGO staging was significantly lower (P<0.05).

**Conclusion:**

We have established two prognostic nomograms for recurrence and long-term survival in patients with OCCC after primary treatment in a large Chinese center and validated them in patients from the same center. This tool used variables specifically related to OCCC and was more accurate than the FIGO system. It is relatively easy to use in clinic for patient counseling, postoperative management, and follow-up for individual patients.

## Introduction

Ovarian clear cell carcinoma (OCCC) is a rare subtype of epithelial ovarian cancer in the United States, while it represents 11.1% of epithelial ovarian cancer in Asians (1–3). In general, OCCC is thought to have different biological characteristics from other types of epithelial ovarian cancer. OCCC is found to arise from endometriosis or clear cell adenofibroma, and is likely to be diagnosed at early-stage, with a relatively good prognosis (2, 4–6). However, advanced-stage OCCC is found to have poor prognosis due to its resistance to chemotherapy (1, 7).

Nomograms have been developed to be an alternative standard for cancer prognosis in recent years (8, 9). Several nomograms have been established on epithelial ovarian cancer (10, 11). However, most nomograms on epithelial ovarian cancer are based on mixed histology of epithelial ovarian cancer, with high-grade serous ovarian cancer being the most common type. Predictors of different types of epithelial ovarian cancer are different and there is rare attempt to establish a nomogram especially on OCCC.

Considering the relative high incidence of OCCC in Asians, the present study enrolled OCCC patients treated at Department of Obstetrics and Gynecology in Peking Union Medical College Hospital, a large ovarian carcinoma center in China and the aims of the present study were to identify significant indicators and develop nomograms for progression-free survival (PFS) and overall survival (OS) for patients with OCCC in China. In addition, the present study compared the predictive accuracy with the currently used International Federation of Gynecology and Obstetrics (FIGO) staging system.

## Methods

### Participants

Patients diagnosed with pure OCCC and treated in Peking Union Medical College Hospital, Beijing, China from 1982-9 to 2016-11 were enrolled in our study. Patients with early stage (stage I and II) OCCC have undergone radical surgical staging (RSS) including total abdominal hysterectomy, bilateral salpingo-oophorectomy, systematic pelvic and para-aortic lymphadenectomy, and radical omentectomy. Patients with advanced OCCC (stage III and IV) have received either primary debulking surgery (PDS), followed by platinum and taxane chemotherapy, or neoadjuvant chemotherapy (NACT), followed by interval debulking surgery (IDS) and subsequent chemotherapy. Although optimal debulking (less than 1 cm in maximum diameter of residual tumor) or complete resection (no residual tumor) is desirable, those with unresectable tumors received suboptimal debulking surgery. In terms of surgical approaches, both laparotomy and laparoscopy were performed by our center.

They all received adjuvant platinum-based chemotherapy after primary surgery. Women who did not received surgery, women who did not received platinum-based chemotherapy after primary surgery, women with mixed type of ovarian carcinoma and women with concurrent cancer other than ovarian cancer were excluded. All participants provided written informed consent. The study was approved by the Ethics Committee of Peking Union Medical College Hospital (S-K903).

For the assessment of survival, PFS was defined as the time from diagnosis to the date of disease progression or end of the study. OS was defined as the time from the date of initial diagnosis to the date of cancer-related death or loss of follow-up.

### Variables

Preoperative demographics and clinical information such as: diagnosed age, body mass index, carbohydrate antigen 125 before operation, endometriosis, thrombosis, past medical history, operative procedure, postoperative chemotherapy (taxane and platinum based chemotherapy), the FIGO staging (according to the 2014 the FIGO staging for ovarian, fallopian tube and peritoneal cancer), macroscopical information (maximal tumor diameter, bilateral or unilateral of tumor, and the residual of the tumor), microscopical information (histologic type, the presence of lymph node metastasis, peritoneal cytology, and malignant ascites or washing), and mode of recurrence and death were collected from medical records and clinical follow-up visit. We also analyzed platinum resistance: Patients who showed recurrence in less than 6 months after completion of primary treatment were classified into platinum resistant group, while patients who relapsed 6 months or more or those who completed taxane and platinum-based chemotherapy and did not experience disease recurrence for at least 6 months of the follow-up period were classified into platinum sensitive group. Patients with insufficient observable time to determine platinum sensitivity were also excluded.

### Statistical analyses

Categorical variables were compared using the χ2 test or Fisher’s exact test. Continuous variables were compared using the t test or Mann-Whitney U test for variables with an abnormal distribution. In the univariate analysis, crude analyses were performed to identify potential risk factors. Then, multivariate analyses with backward procedures were used to select the best-fit model. A statistical significance level of 0.20 was used to select variables into the model.

Stage I and II were combined and Stage III and IV were combined in the FIGO staging due to the small sample size of Stage II and Stage IV patients. Platinum resistance was considered a variable in the analyses of OS prognosis.

Patients diagnosed from 1982-9 to 2011-12 were classified as the training group and patients diagnosed from 2012-1 to 2016-11 were classified as the validation group. Nomograms were constructed based on the results of multivariate analysis to predict PFS, and OS from the training group. The performance of the nomogram was measured by concordance index and calibration curve using a bootstrapped sample. Model validation was performed using bootstrap resampling to quantify the overfitting of our modeling strategy and predict future performance of the model. Then, internal validation was performed on the validation group. Statistical analyses were performed using the package in R version 2.14.1 (http://www.r-project.org/).

## Results

A total of 358 patients were included in the study, with 247 (69.0%) enrolled in the training group and 111 (31.0%) recruited in the validation group. The mean diagnosed age was 49.5 ± 10.5 years. Of these patients, 13.69% had thrombosis and 37.63% had endometriosis before operation. The training group and validation group had no significant difference in age, body mass index, thrombosis, endometriosis, surgical approaches and surgical procedures. The validation group underwent less advanced-stage patients and less residual tumor than the training group (**Table 1**).

**Table 1:** Patients’ characteristics.

Variables	All patients (n = 358)	Training group(n = 247)	Validation group(n = 111)	P value
Age of diagnosis/y (mean ± standard deviation)	49.5 ± 10.5	50.0 ± 10.5	48.4 ± 10.4	0.197
Body mass index of diagnosis/kg/m2 (mean ± standard deviation)	22.6 ± 3.1	25.5 ± 7.2	22.5 ± 2.7	0.704
Thrombosis n(%)				0.119
No n (%)	309 (86.31%)	208 (84.21%)	101 (90.99%)	
Yes n (%)	49 (13.69%)	39 (15.79%)	10 (9.01%)	
Endometriosis n(%)				0.726
No n (%)	179 (62.37%)	111 (61.33%)	68 (64.15%)	
Yes n (%)	108 (37.63%)	70 (38.67%)	38 (35.85%)	
Elevated Carbohydrate antigen 125 n(%)				0.010
No n (%)	159 (44.41%)	107 (43.32%)	52 (46.85%)	
Yes n (%)	199 (55.59%)	140 (56.68%)	59 (53.15%)	
Surgical approaches				0.405
Laparotomy n(%)	326 (91.06%)	227 (91.90%)	99 (89.19%)	
Laparoscopy n(%)	32 (8.94%)	20 (8.10%)	12 (10.81%)	
Surgical procedures for patients with advanced stages				0.006
Primary debulking surgery n(%)	72 (52.17%)	59 (54.63%)	13 (43.33%)	0.273
Interval debulking surgery n(%)	66 (47.83%)	49 (45.37%)	17 (56.67%)	
Tumor side				0.006
Unilateral n(%)	265 (74.86%)	171 (70.37%)	94 (84.68%)	
Bilateral n(%)	89 (25.14%)	72 (29.63%)	17 (15.32%)	
The International Federation of Gynecology and Obstetrics staging				0.004
I n(%)	196 (54.75%)	122 (49.39%)	74 (66.67%)	
II n(%)	24 (6.7%)	17 (6.88%)	7 (6.31%)	
III n(%)	120 (33.52%)	97 (39.27%)	23 (20.72%)	
IV n(%)	18 (5.03%)	11 (4.45%)	7 (6.31%)	
Maximal diameter/cm (mean ± standard deviation)	11.1 ± 6.7	11.3 ± 5.6	10.8 ± 8.5	0.529
Lymph node metastasis				0.082
Negative n (%)	287 (82%)	193 (79.42%)	94 (87.85%)	
Positive n (%)	63 (18%)	50 (20.58%)	13 (12.15%)	
Residual of the tumor n(%)				0.002
Negative n (%)	274 (76.54%)	177 (71.66%)	97 (87.39%)	
Positive n (%)	84 (23.46%)	70 (28.34%)	14 (12.61%)	
Ascites/malignant washing n(%)				0.261
Negative n (%)	296 (82.68%)	200 (80.97%)	96 (86.49%)	
Positive n (%)	62 (17.32%)	47 (19.03%)	15 (13.51%)	
Peritoneal cytology n(%)				0.002
Negative n (%)	228 (63.69%)	144 (58.3%)	84 (75.68%)	
Positive n (%)	130 (36.31%)	103 (41.7%)	27 (24.32%)	
Distant metastasis n(%)				0.631
Negative n (%)	340 (94.97%)	236 (95.55%)	104 (93.69%)	
Positive n (%)	18 (5.03%)	11 (4.45%)	7 (6.31%)	

At the end of this study, recurrence occurred in 130 (36.3%) patients, while 55 (15.4%) patients were lost to follow-up, and 173 (48.3%) patients remained progression-free. 61 (17.0%) patients had died of OCCC, 34 (9.5%) patients were lost to follow-up, and 263 (73.5%) patients remained alive.

**Figure 1:**
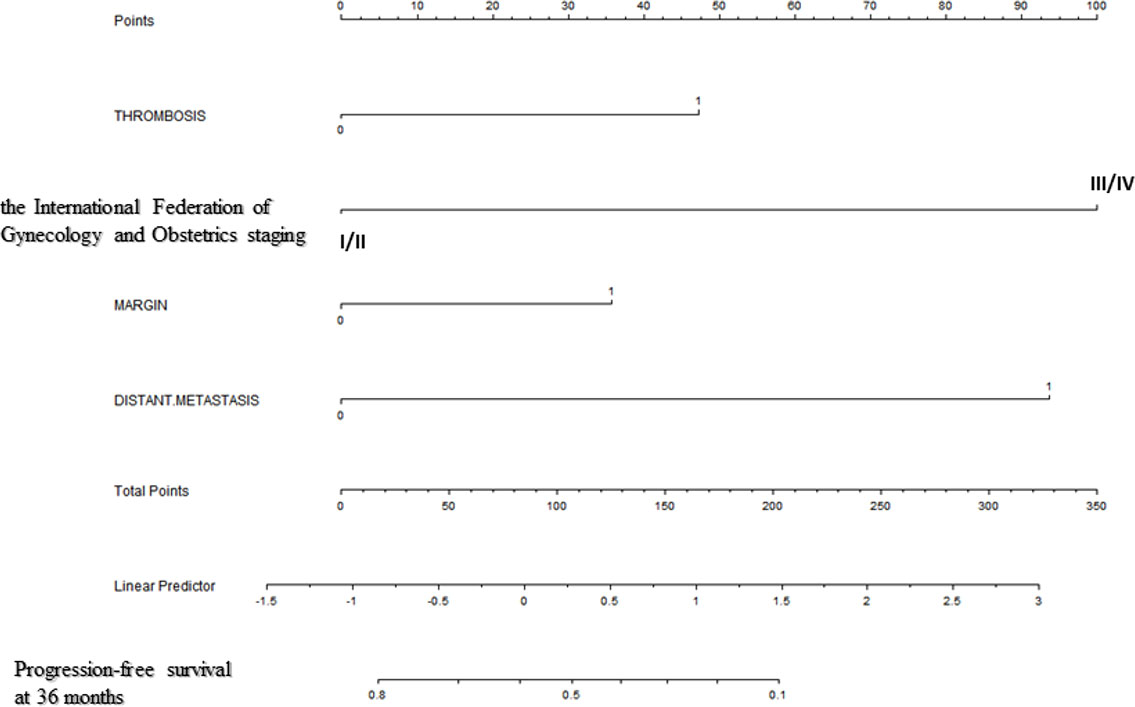
A Nomogram for Predicting Progression-free Survival of Patients With Resectable Ovarian Clear Cell Carcinoma After Primary Treatment. To calculate predicted progression-free survival, an individual patient’s value is located on each variable axis, and a straight line is drawn upward to the “Points” row to determine the points associated with each factor. After summing the total points, one locates the appropriate total point number and draws a straight line from this to the bottom rows to determine the patient’s predicted survival probability. (For each variable: Thrombosis: 0=no thrombosis, 1=exist; The International Federation of Gynecology and Obstetrics staging: I/II=stage I/II, III/IV=stage III/IV; Residual of the tumor: 0=negative, 1=positive; Distant metastasis: 0=no distant metastasis, 1=positive distant metastasis).

**Figure 2:**
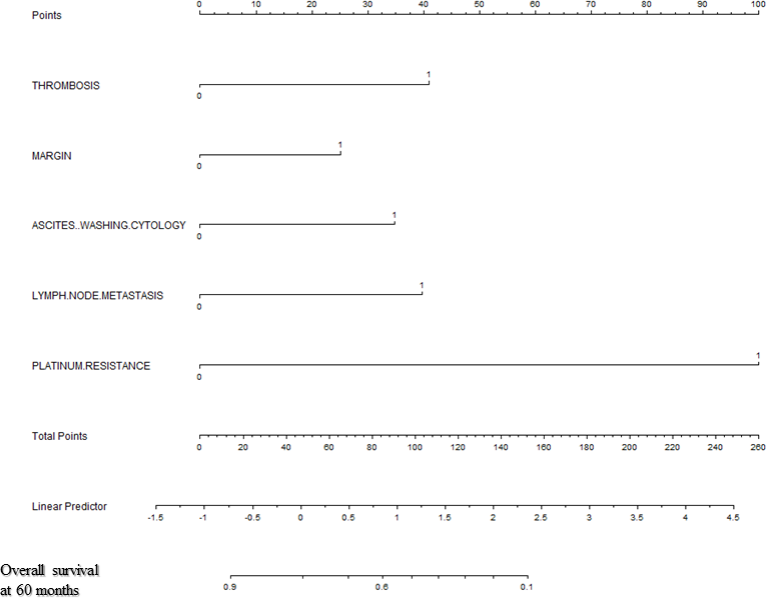
A Nomogram for Predicting Overall Survival of Patients With Resectable Ovarian Clear Cell Carcinoma After Primary Treatment. To calculate predicted overall survival, an individual patient’s value is located on each variable axis, and a straight line is drawn upward to the “Points” row to determine the points associated with each factor. After summing the total points, one locates the appropriate total point number and draws a straight line from this to the bottom rows to determine the patient’s predicted survival probability. (For each variable: Thrombosis: 0=no thrombosis, 1=exist; Residual of the tumor: 0=negative, 1=positive; Malignant ascites/washing: 0=no malignant ascites/washing, 1= malignant ascites/washing; Lymph node metastasis: 0=negative lymph node metastasis, 1=positive lymph node metastasis; Platinum resistance: 0=platinum sensitive, 1=platinum resistant).

Backward stepwise selection in Cox proportional hazards regression model identified several variables that were the most associated with PFS and OS, respectively (**Tables 2**, **3**). Then, nomograms were developed using the selected variables (**Figures 1**, **2**). The nomogram for the prediction of PFS included thrombosis, the FIGO staging, residual of the tumor, distant metastasis. Each factor was assigned a weighted point and patients with a higher total score had a higher risk for recurrence. Discrimination of the model measured by the Harrell concordance index was 0.738 (**Figure 1**). By the same algorithm, the nomogram for predicting OS was developed. The nomogram consisted of thrombosis, lymph node metastasis, residual of the tumor, malignant ascites/washing, and platinum resistance, with a Harrell concordance index of 0.835 (**Figure 2**).

**Table 2:** Progression-free survival of patients with ovarian clear cell carcinoma.

Variables	Univariate analysis			Multivariate analysis		
	Harzard ratio	95% Confidence interval	P value	Harzard ratio	95% Confidence interval	P value
Age of diagnosis/y	1.01	0.99-1.03	0.332			
Body mass index of diagnosis/kg/m2	1.04	0.89-1.21	0.655			
Thrombosis	2.20	1.41-3.44	<0.001	1.64	1.02-2.66	0.042
Endometriosis	1.27	0.73-2.2	0.395			
Elevated Carbohydrate antigen 125	4.03	2.06-7.88	<0.001			
Surgical approaches			0.765			
Laparotomy	Reference	Reference	Reference			
Laparoscopy	1.01	0.65-1.33	0.765			
Surgical procedures for patients with advanced stages			0.685			
Primary debulking surgery	Reference	Reference	Reference			
Interval debulking surgery	1.05	0.78-1.21	0.685			
Bilateral tumor side	2.20	1.46-3.32	<0.001			
The International Federation of Gynecology and Obstetrics staging			<0.001			<0.001
I/II	Reference	Reference	Reference	Reference	Reference	Reference
III/IV	4.41	2.94-6.6	<0.001	3.41	2.03-5.73	<0.001
Maximal diameter/cm	1.00	0.96-1.05	0.838			
Lymph node metastasis	2.92	1.86-4.57	<0.001			
Residual of the tumor	3.73	2.47-5.62	<0.001	1.7	0.99-2.91	0.053
Positive ascites/malignant washing	2.27	1.46-3.52	<0.001			
Positive peritoneal cytology	4.19	2.81-6.25	<0.001			
Distant metastasis	6.03	2.83-12.87	0.000	3.18	1.44-7.02	0.004

**Table 3:** Overall survival of patients with ovarian clear cell carcinoma.

Variables	Univariate analysis			Multivariate analysis		
	Harzard ratio	95% Confidence interval	P value	Harzard ratio	95% Confidence interval	P value
Age of diagnosis/y	1.02	1-1.05	0.084			
Body mass index of diagnosis/kg/m2	0.69	0.4-1.18	0.173			
Thrombosis	2.65	1.53-4.6	<0.001	2.98	1.4-6.34	0.005
Endometriosis	1.11	0.58-2.13	0.752			
Elevated Carbohydrate antigen 125	5.23	2.23-12.25	<0.001			
Surgical approaches			0.694			
Laparotomy	Reference	Reference	Reference			
Laparoscopy	1.04	0.78-1.37	0.694			
Surgical procedures for patients with advanced stages			0.324			
Primary debulking surgery	Reference	Reference	Reference			
Interval debulking surgery	1.02	0.89-1.13	0.324			
Bilateral tumor side	4.00	2.4-6.65	<0.001			
The International Federation of Gynecology and Obstetrics staging			<0.001			
I/II	Reference	Reference	Reference			
III/IV	<0.001	<0.001	<0.001			
Maximal diameter/cm	1.00	0.95-1.05	0.949			
Lymph node metastasis	4.69	2.75-8.01	<0.001	2.47	1.12-5.42	0.025
Residual of the tumor	8.30	4.92-14	<0.001	1.73	0.8-3.74	0.162
Positive ascites/malignant washing	4.32	2.57-7.25	<0.001	1.95	0.93-4.08	0.076
Positive peritoneal cytology	5.32	3.12-9.06	<0.001			
Distant metastasis	3.40	1.44-8.02	0.005			
Neoadjuvant chemotherapy	2.73	1.56-4.76	<0.001			
Platinum resistance	1.84	0.99-3.4	0.053	10.27	4.5-23.45	<0.001

**Figure 3:**
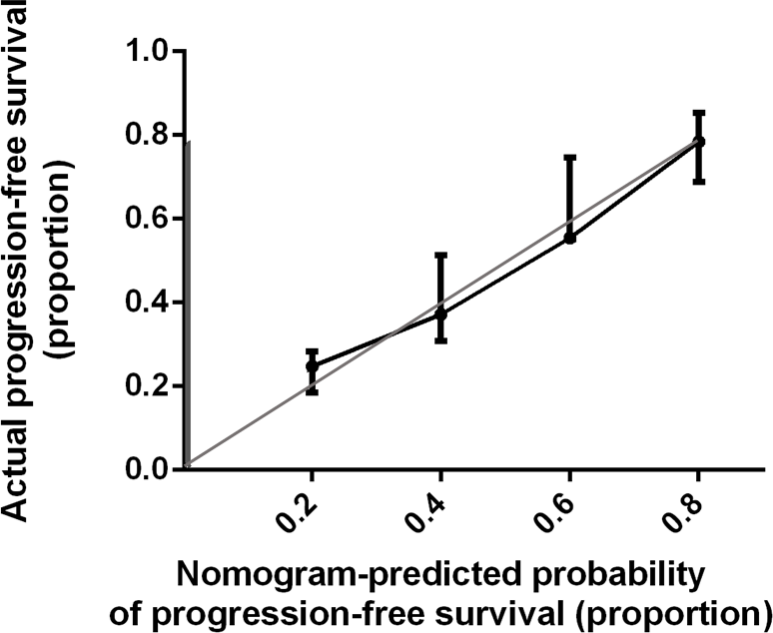
Calibration Plot Comparing Predicted and Actual Progression-free Survival Probabilities. The calibration curve for predicting patient progression-free survival is stated in **Figure 3**. Nomogram-predicted probability of progression-free survival is plotted on the x-axis; actual progression-free survival is plotted on the y-axis. Thin gray line represents the reference line.

**Figure 4:**
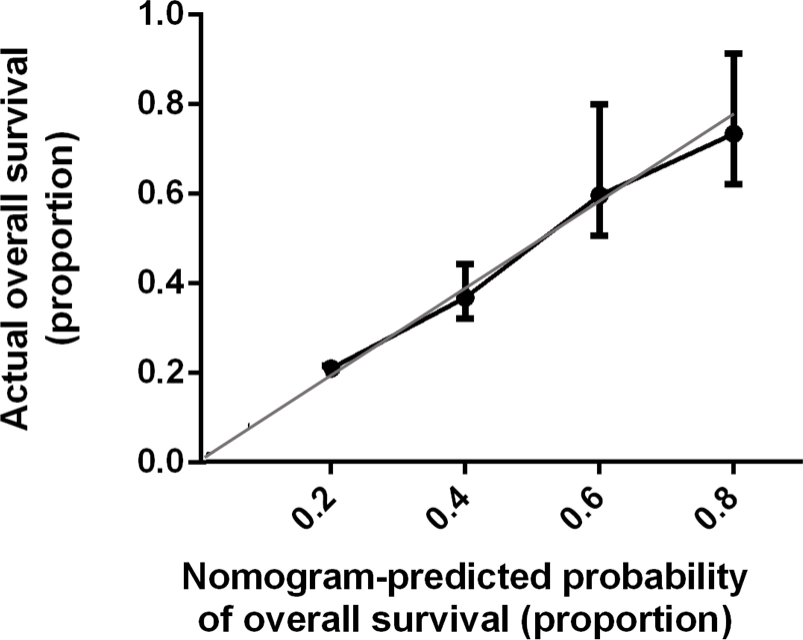
Calibration Plot Comparing Predicted and Actual Overall Survival Probabilities. The calibration curve for predicting patient overall survival is stated in **Figure 4**. Nomogram-predicted probability of overall survival is plotted on the x-axis; actual overall survival is plotted on the y-axis. Thin gray line represents the reference line.

Bootstrap validation of the model with 500 iterations revealed minimal evidence of model overfit. The calibration plot of the models showed good predictive accuracy (**Figures 3**, **4**). In the validation cohort, the concordance index of were 0.775 and 0.807 for predicting PFS and OS, respectively.

In comparison, the concordance statistics for PFS and OS of OCCC based on the FIGO staging were 0.715 and 0.727, respectively. The concordance indice of the FIGO staging for OS was significantly lower than that of the nomogram (P < 0.05) while the concordance indice for PFS showed no significance.

## Discussion

In the present study, we successfully established nomograms for predicting PFS and OS of patients with OCCC in a large ovarian cancer center in China. The Harrell concordance indexes of these models were 0.873, 0.738 and 0.835, respectively.

OCCC is thought to have different biological characteristics from other types of epithelial ovarian cancer while there are rare attempts to establish a nomogram especially on OCCC. Therefore, we successfully established nomograms using special prognostic factors in this type of ovarian cancer. In addition, it was firstly demonstrated that our nomogram was more accurate than the FIGO system. It is relatively easy to use in clinic for patient counseling, postoperative management, and follow-up for individual patients.

Such predictive nomograms are of great clinical value with the era of precision medicine. In clinic, these indicators were found to be of great significance for prediction. For example, if an OCCC patient comes to the clinic after primary treatment, the doctor can directly tell him/her the probability of recurrence and prediction of OS using our nomograms. In addition, this model will have a great effect on guiding the next treatment plan in clinical work. If a patient has a high risk of recurrence, more aggressive treatments such as intraperitoneal chemotherapy and more frequent follow-up might be recommended. Therefore, our nomogram makes our evaluation system applicable to patients who have undergone surgery, and can also calculate the prognosis of patients at follow-up.

Several nomograms for predicting survival prognosis of epithelial ovarian cancer have been developed. Obermair et al. (12) introduced a nomogram to predict the recurrence probability in patients with borderline ovarian tumors, while Meurs et al. (13) developed models to predict the risk of recurrence free survival for various types of ovarian tumor. Several studies also (14–16) published nomograms predicting survival for epithelial ovarian cancer. Thus, nomograms for epithelial ovarian cancer have been developed in multiple populations. However, nomograms for OCCC are sparse. One reason might be due to the low incidence in Western women (1). In addition, OCCC has some special characteristics such as the large amount of endometriosis complications, the high rate of thromboembolic complications, and the poor response to chemotherapy, which are different from other subtypes of epithelial ovarian cancer and might influence the prognosis of OCCC (4, 5). Some studies evaluated OCCC (14, 15) with small sample size, while Chen et al. established a nomogram for patients with OCCC based on the Surveillance, Epidemiology, and End Results database (17). However, these studies rarely considered factors specially related with OCCC. Our nomograms for PFS and OS of OCCC combined a series of prognostic factors, such as thrombosis and platinum resistance, which were not incorporated in the FIGO system and previous Chen’s research (17). Widely used prognostic systems like the FIGO classification include a limited number of tumor-related variables and it is unknown that whether additional risk factors are of important prognostic values.

In our study, thrombosis was found to be a prognostic factor for OCCC for both PFS and OS, and remained an independent prognostic factor for PFS and OS. The risk of thromboembolic events is demonstrated to higher in OCCC than other histologic subtypes of epithelial ovarian cancer (18). Elena et al. in 2013 demonstrated that thromboembolic events during OCCC primary treatment were associated with a significantly higher risk of cancer recurrence and death (19), which was consistent with our results. Tissue factor is a transmembrane glycoprotein that serves as a physiologic initiator of coagulation and implicates in tumor growth, metastasis, and angiogenesis (20, 21). It was found to be a modulator in thromboembolic events in epithelial ovarian cancer (22). The hypothesis that a paracrine circuit involving thrombosis could lead to more aggressive tumor biology might also contribute to this increased risk (19). Thus, we included thrombosis in both PFS and OS nomogram establishment.

Platinum resistance was another factor which was independently associated with OS for our OCCC patients. A lot of retrospective studies showed that the response rate of OCCC to traditional platinum-based chemotherapy was significantly lower than serous adenocarcinoma (23–25). A Korea group (15) in 2019 also considered platinum resistance as a prognostic factor in developing nomogram for OS in epithelial ovarian cancer. Therefore, considering the high rate of platinum resistance in OCCC and its prognostic value, we also included it in the development of OS nomogram.

In addition, we have explored the influence of surgery approaches and surgery approaches on PFS and OS. No significant difference was found in OS and PFS between patients undergoing laparotomy and minimally invasive surgery, which was consistent with previous research (26, 27). This indicated that both laparotomy and laparotomy would be applicable in the surgery of ovarian cancer and would not influence the survival of patients. We also explored the influence of PDS vs. IDS in the sub group of patients with advanced OCCC and found that none of the two investigated procedures has proven to be superior in terms of OS and PFS, which was also consistent with the previous research (28, 29).

Furthermore, our nomograms were comparable with the FIGO staging. The concordance statistics of the nomogram was even significantly higher than that of the FIGO staging for OS assessment. Thus, our nomograms had great prognostic value. In this study, we established two prognostic nomograms for recurrence and long-term survival after primary treatment in a large center in China and validated them in patients from the same center. The nomogram is relatively easy to use in clinic for predicting the survival rate for individual patients.

The study has some limitations. First, this study was conducted in a single institution. Further studies in multicenter should be constructed. Second, it was a retrospective data analysis. Although we performed internal validation with a good result, future externally validation is needed. In future research, we would combine a multicenter data analysis to verify the accuracy and usefulness of our model and to increase the validity of the data. Last but not least, it was not well-known by obstetrician and gynecologist. An app which embedded this nomogram might be designed in the future to make it easier and faster to provide prognostic information.

## Data availability statement

The raw data supporting the conclusions of this article will be made available by the authors, without undue reservation.

## Ethics statement

The study was approved by the Ethics Committee of Peking Union Medical College Hospital (S-K903). The patients/participants provided their written informed consent to participate in this study.

## Author contributions

JL: Conceptualization; Data curation; Formal analysis; Investigation; Methodology; Software; Visualization; Writing–original draft. DC: Conceptualization; Funding acquisition; Methodology; Project administration; Resources; Software; Supervision; Validation; Visualization; Writing–review & editing. All authors read and approved the final manuscript.

## Acknowledgments

The authors would like to thank Dr. Yan You in pathology department of Peking Union Medical College Hospital, for helping the confirmation the diagnosis of the patients. And the authors would like to thank Dr Xiaoshuang Zhou, Shuang Ye, Dr Xiao Huo and Dr Hengzi Sun for helps.

## Conflict of interest

The authors declare that the research was conducted in the absence of any commercial or financial relationships that could be construed as a potential conflict of interest.

## Publisher’s note

All claims expressed in this article are solely those of the authors and do not necessarily represent those of their affiliated organizations, or those of the publisher, the editors and the reviewers. Any product that may be evaluated in this article, or claim that may be made by its manufacturer, is not guaranteed or endorsed by the publisher.
